# Evaluation of the optimal combinations of modulation factor and pitch for Helical TomoTherapy plans made with TomoEdge using Pareto optimal fronts

**DOI:** 10.1186/s13014-015-0497-2

**Published:** 2015-09-17

**Authors:** Geert De Kerf, Dirk Van Gestel, Lobke Mommaerts, Danielle Van den Weyngaert, Dirk Verellen

**Affiliations:** Department of Radiotherapy, University Radiotherapy Antwerp (URA), Antwerp, Belgium; Present address: Department of Radiotherapy, Iridium Cancer Network, GZA Sint-Augustinus, Oosterveldlaan 24, 2610 Wilrijk, Antwerp Belgium; Present address: Department of Radiotherapy, Institut Jules Bordet, Université Libre de Bruxelles, Brussels, Belgium; Radiotherapy UZ Brussel, Faculty of Medicine and Pharmacy Vrije Universiteit Brussel, Brussels, Belgium

## Abstract

**Background:**

Modulation factor (MF) and pitch have an impact on Helical TomoTherapy (HT) plan quality and HT users mostly use vendor-recommended settings. This study analyses the effect of these two parameters on both plan quality and treatment time for plans made with TomoEdge planning software by using the concept of Pareto optimal fronts.

**Methods:**

More than 450 plans with different combinations of pitch [0.10–0.50] and MF [1.2–3.0] were produced. These HT plans, with a field width (FW) of 5 cm, were created for five head and neck patients and homogeneity index, conformity index, dose-near-maximum (D2), and dose-near-minimum (D98) were analysed for the planning target volumes, as well as the mean dose and D2 for most critical organs at risk. For every dose metric the median value will be plotted against treatment time. A Pareto-like method is used in the analysis which will show how pitch and MF influence both treatment time and plan quality.

**Results:**

For small pitches (≤0.20), MF does not influence treatment time. The contrary is true for larger pitches (≥0.25) as lowering MF will both decrease treatment time and plan quality until maximum gantry speed is reached. At this moment, treatment time is saturated and only plan quality will further decrease.

**Conclusion:**

The Pareto front analysis showed optimal combinations of pitch [0.23–0.45] and MF > 2.0 for a FW of 5 cm. Outside this range, plans will become less optimal. As the vendor-recommended settings fall within this range, the use of these settings is validated.

## Introduction

Differences between most common planning systems have been extensively investigated in planning studies using statistical [1–9]. However, this method is susceptible to planner’s experience and consequently results can be biased [10]. New concepts, like Pareto optimal fronts, can be used as a more robust plan comparison tool as it gives a more objective evaluation of contradictory objectives [11].

A Pareto front is the collection of Pareto optimal solutions. Because inverse planning makes use of contradictory objectives, high and uniform target dose on the one hand and sparing of organs at risk (OARs) on the other, a Pareto front analysis is well suited for showing optimal combinations in a multi-objective optimization [12–15]. To create a Pareto front, a database of Pareto optimal plans must be made. A Pareto optimal solution is obtained when one objective cannot be improved without deterioration in another. Therefore, the concept can be directly used in inverse treatment planning (as implemented by RaySearch [RaySearch Laboratories, Stockholm, Sweden] in their multi criteria optimization software, called RayStation).

In contrast to a multi objective optimization, this study only uses the concept of Pareto optimal fronts for analysis to investigate the impact of Helical TomoTherapy’s (HT) specific plan parameters, pitch and *modulation factor* (MF), on plan quality. The complicated head and neck (H&N) region, with radiosensitive OARs situated close to rather radio resistant target volumes, represents an ideal platform for this study.

The HT planning software requires unique parameters to be set (field width, pitch and MF) that influences plan quality. The *field width* (FW) is defined as the axial thickness of the fan beam and a smaller FW allows more modulation in cranial-caudal direction. The pitch is defined as the couch travel distance for a complete gantry rotation relative to the axial beam width at the axis of rotation. Usually, a pitch of 0.86/n, where n is an integer, is used to reduce the thread effect [16]. The MF is defined as the maximum leaf opening time divided by the average (non-zero) leaf opening time and it is an estimate of plan complexity. The planning MF can be set as an input in the planning system. The actual MF indicates the required complexity of the plan and is limited by the planning MF. Only planning MF is considered in this study. Most common settings for a H&N treatment are: FW = 2.5 cm, pitch = 0.287, MF = ≈3.0 [16]. For treatment planning, the continuously rotating fan beam with binary multi leaf collimator (MLC is open or closed) is discretized in 51 beams per rotation. Per angle dose is delivered via the concept of beamlets.

The latest software version of the HT planning system has the TomoEdge Dynamic Jaws option allowing the jaws to open dynamically at the cranial and caudal edges of the target volume. As TomoEdge creates for every FW (2.5 cm or 5 cm) a cranio-caudal dose gradient at the beginning and end of the PTV as sharp as a 1 cm field, treatment times can be reduced by selecting a larger FW without increasing the integral dose to the patient [17, 18].

This study explores the influence of the HT-specific parameters (pitch and MF) on plan quality and treatment time for a FW of 5 cm when fixed optimization constraints are used. The concept of Pareto optimal fronts is used for analysis and this analysis will explore whether the widely used vendor-recommended settings are optimal for treatment planning.

## Materials and methods

### Treatment planning

For five patients with locally advanced oropharyngeal cancer, plans with varying combinations of pitch and MF have been made. CT scans and contours were adopted from a previous study [9].

For every patient a set of HT plans was made using a HT Planning Station – TomoHDA Version 2.0.0. This set contains one reference plan and plans for creating the Pareto optimal front. The reference plan (REF5cm) was fully optimized to our current practice using standard settings (FW = 5 cm, pitch = 0.430 and MF = 2.8). To obtain plans for the Pareto front analysis, this reference plan and its optimization constraints were copied and pitch and MF were varied in nine steps within the range [pitch = 0.10–0.50] and ten steps within the range [MF = 1.2–3.0], respectively. This resulted in 90 treatment plans per patient, each with a unique combination of pitch and MF. The chosen ranges of pitch and MF are limited by our clinically used settings. For comparison, a second reference plan (REF2.5 cm) was produced (FW = 2.5 cm, pitch = 0.287 and MF = 2.8).

For each plan used to help generate the Pareto optimal front the same constraints were used during an optimization process of 200 iterations, which differs from a full multi-objective optimization. No interaction during the optimization process was allowed in order to avoid planner induced biases, except for the REF plans.

The dose distributions were calculated with voxel-less optimization (VoLO) [19], using a convolution/superposition algorithm [20] and a normal grid size of 3.8 mm × 3.8 mm.

### Prescription

A simultaneous integrated boost technique was planned to deliver a dose of 69.12 Gy (2.16 Gy/fraction) in 32 fractions to the therapeutic planning target volume ‘PTV69’ and a dose of 56 Gy (1.75 Gy/fraction) to the elective ‘PTV56’. Each reference plan was set to respect the prescription guidelines of the International Commission on Radiation Units and Measurements (ICRU) report 83 [21], and the dose to all OARs had to be kept as low as possible.

### Data reporting

For each patient multiple combinations of pitch and MF were generated. Several specific and critical doses and irradiated volumes of different OARs (mean dose [Dmean] and dose-near-maximum [D2 %, dose received by 2 % of the tissue]) and PTVs (homogeneity index [HI, (D2 %-D98 %)/D_prescribed_], conformity index [CI, V95 %D_prescribed(body)_/V95 %D_prescribed(PTV)_], dose-near-minimum [D98 %, dose received by 98 % of the tissue] and D2 %) were calculated with an in-house written Matlab program. Treatment times were also reported.

### Data analysis

For these five H&N patients, representing a class solution for each unique combination of pitch and MF, a median value was calculated for every reported OAR/PTV dose and index (HI, CI, D98, D2 and/or Dmean). Out of this inter patient median value different Pareto-like fronts have been created to show the influence of MF and pitch on both treatment time and the chosen dose value.

## Results

**Fig. 1:**
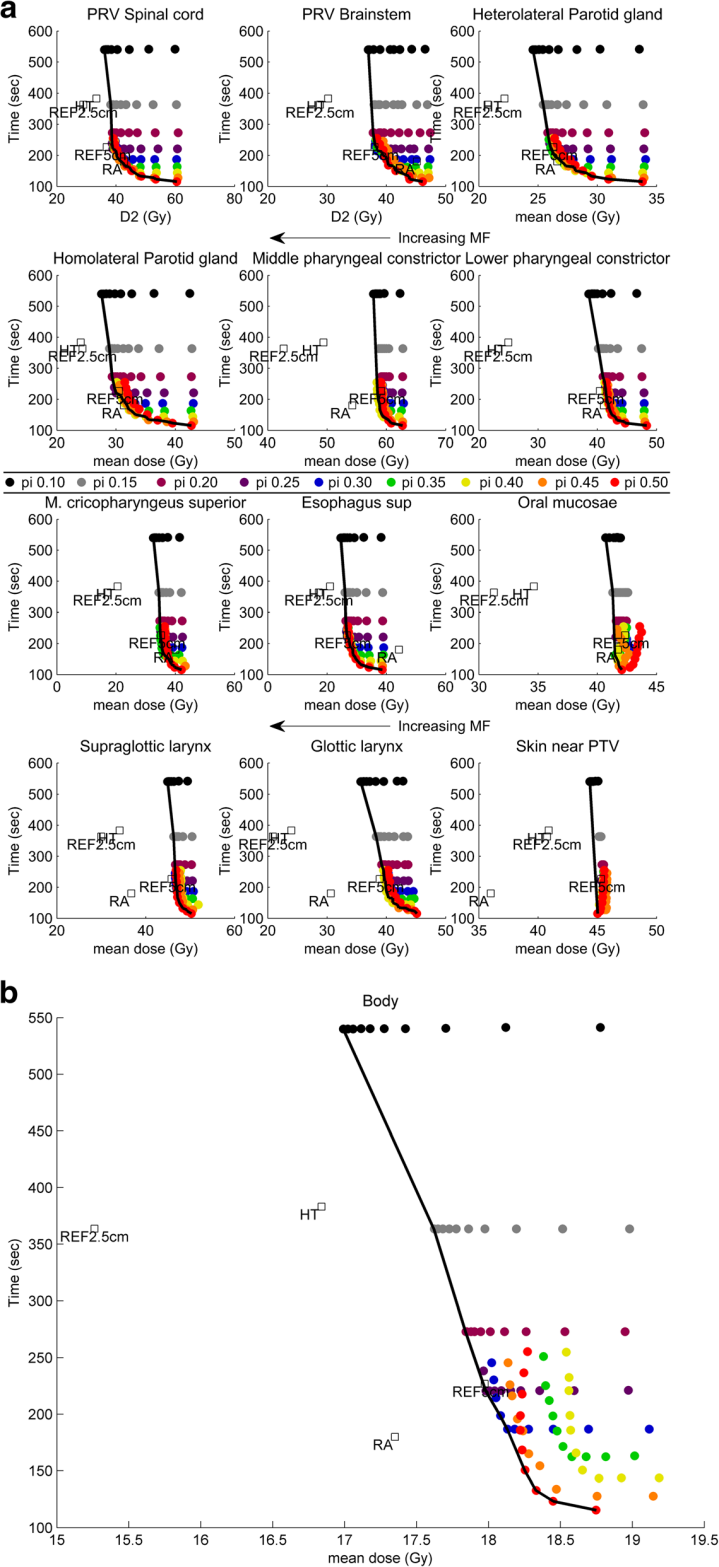
**a**: OAR Pareto fronts (from left to right and top to bottom) of D2 for PRV cord and PRV brainstem and for the mean dose of oral mucosa, heterolateral parotid gland, homolateral parotid gland, oesophagus, lower pharyngeal constrictor, middle pharyngeal constrictor, cricopharyngeal muscle, glottic larynx, supraglottic larynx and skin near PTV. Every colour represents a single pitch and per colour the subsequent dots (dot = median value) represent, from right to left, an increasing MF. Dots lying on top of the black solid line are Pareto optimal.**b**: Pareto front of Body mean dose.

**Fig. 2:**
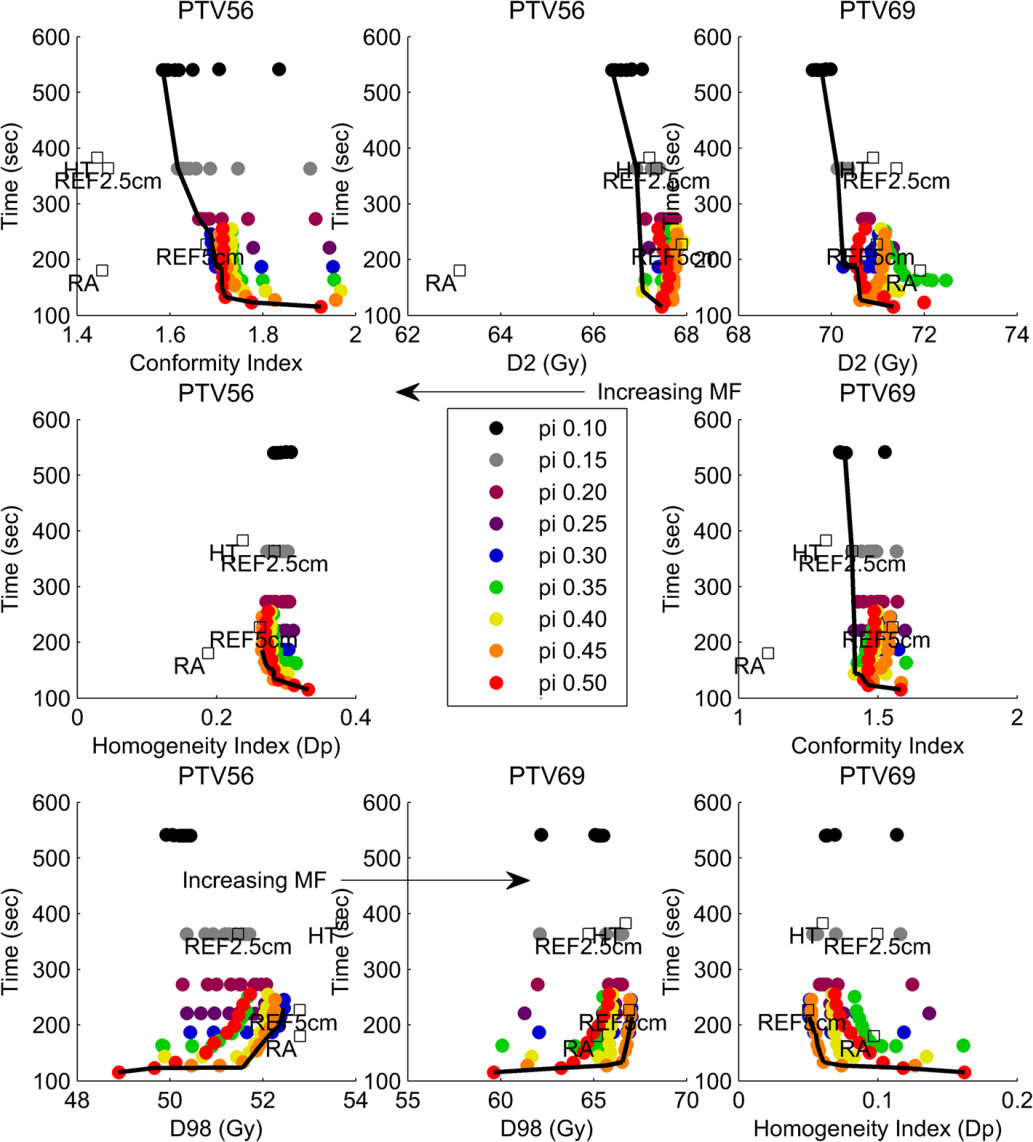
Target volume Pareto fronts of conformity index, homogeneity index, D2 and D98 for PTV56 and PTV69. Every colour represents a single pitch and per colour the subsequent dots (dot = median value) represent a change in MF. The arrow points at increasing MF (for D98 the arrow is reversed). Dots lying on top of the black solid line are Pareto optimal

The Pareto fronts of the OARs and the target volumes as shown in Figs. 1a, 1b and 2 were calculated out of 450 plans (90 plans produced per patient, each with a different combination of pitch and MF). Only the data points that lie on top of the black Pareto line approximate the Pareto optimal front. In addition to the Pareto front, data from a previous study [9], containing both a ‘standard’ HT plan (FW 2.5 cm, pitch 0.287, MF 2.8 for TomoHD, version 1.0.0; HT data point) and a RapidArc plan (2 full arcs, version 8.6.15; RA data point), are added to these graphs as reference points.Figure 1b gives a more detailed view on how data points behave, related to a changing pitch or MF.

### Treatment time

**Fig. 3:**
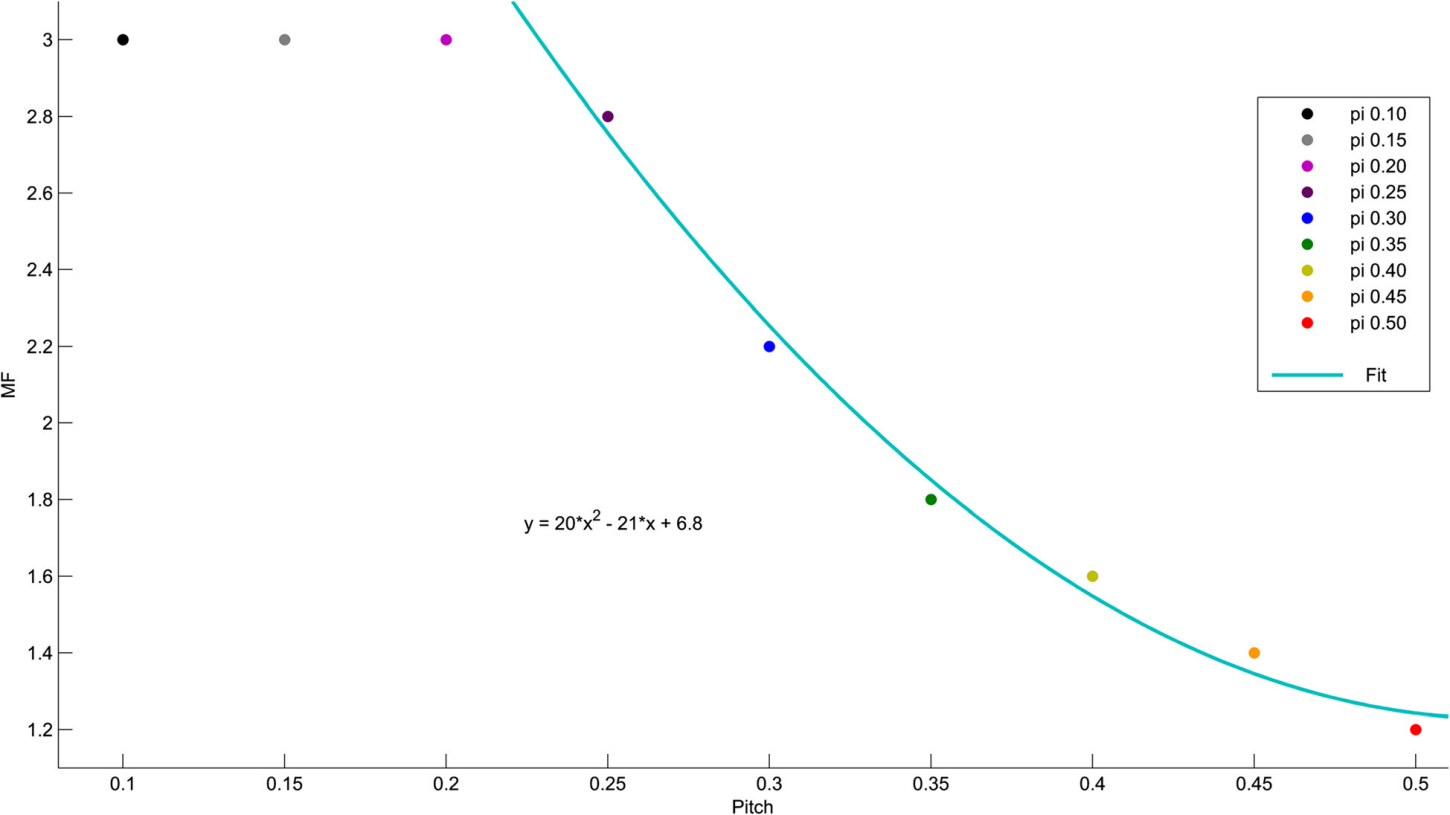
Plot of saturation point (gantry period becomes minimal), as a function of pitch and MF. Quadratic fit through 6 largest pitches

Treatment time was reduced by almost 50 % when a FW of 5 cm was used compared to the (standard) FW of 2.5 cm (Fig. 1), at the cost of a less optimal dose distribution [22]. The REF5cm field has a median treatment time of 3.7 min. For 5 cm fields, treatment time varies with varying pitch and MF. The low pitches (≤0.20) show a constant treatment time, independent of the chosen MF. Higher pitches (≥0.25) have a dual behaviour. Initially, lowering MF will decrease both treatment time and plan quality. Subsequently, treatment time becomes invariable and only dose is further influenced by the varying MF. The saturation point, defined at the transition of these two states, occurs for every pitch at a different MF as plotted in Fig. 3.

### Target volumes

PTV69 has a very homogenous and conformal dose distribution for all plans regardless of pitch, MF or FW (Fig. 2). HI decreases (lower HI = more homogeneous) with decreasing pitch or increasing MF, CI only changes for the very small pitches (0.10–0.25). For pitches larger than 0.25 the conformity index decreases (plans become more and more conformal) with increasing MF until the MF becomes higher than 2.6 or 2.8. D98 also increases with increasing MF for both PTV56 and PTV69. The opposite is true for D2.

### OARs

For all OARs, Pareto fronts can be created, except for the oral mucosa and the skin near PTV, as shown in Fig. 1. Dose to the OARs is influenced by both pitch and MF. The largest dose reduction is introduced when increasing the MF from 1.2 to 1.4 or 1.6. Plans with MF ≤ 1.4 can be classified as clinically unacceptable because of a too high dose to the spinal cord.

## Discussion

Due to the large number of plans, patient specific Pareto fronts with HT planning software is labour intensive. Consequently, the Pareto optimality approach is difficult to use in clinical practice. However, general trends that can be applied in daily routine can be deduced from studies such as this.

A TomoEdge plan (FW of 5 cm) yielded no improvement in plan quality when compared to a plan with a FW of 2.5 cm, as TomoEdge only moves jaws at both edges of the PTV in cranio-caudal direction and not during the rest of the treatment. Consequently, dose penumbra will only be reduced at the outer edges of the treatment volume [18, 23]. During treatment, dose reduction in the OARs is limited to the modulation capabilities of the 5 cm field. Because a plan with a 2.5 cm FW can use more beamlets, and thus more optimization possibilities, than a plan with a 5 cm field when treating the same volume, better sparing of the OARs is possible with a smaller FW.

The smallest pitches (0.10 and 0.15) yield no improvement compared to 2.5 cm fields, as the latter result in faster and better plans (lower doses to all OARs). These low pitches show a constant treatment time, independent of the chosen MF, as the gantry period already reached its minimal value of 12 s. This can be explained by the overshoot in optimization possibilities (eg: for a pitch of 0.10, each voxel within the target volume can be optimized by 510 beamlets). A lower MF will only result in a worse dose distribution and a higher dose to the normal tissue. The latter is also valid for a pitch of 0.20 with the exception of its faster treatment time.

Pitches of 0.25 or higher first show a constant behaviour for small MFs (dots lie on a horizontal line), treatment time is unaffected by an initial increase in MF. Once the minimal gantry period becomes too short to obtain enough modulation for a given MF, treatment time increases with increasing MF. The dose distribution to OARs/PTVs also improves with increasing MF. Below the minimal gantry period, further reduction of MF cannot accelerate gantry speed anymore and consequently treatment time becomes invariable and only dose distribution will vary. A relationship exists defining the minimal MF that should be used for a given pitch. This relationship is shown in Fig. 3 and can be predicted by MF = 20 ∗ pitch^2^ − 21 ∗ pitch + 6.8 with norm of residuals = 0.12 and pitch ∈ {0.25 − 0.50} and shows a plateau at pitch ~0.23. This means that for the widely used pitch of 0.430 [16] (for a 5 cm FW), the optimal MF must be larger than 1.5. Below this value, no further gain in treatment time will occur, hence the dose distribution will only become worse.

Oral mucosa and skin near PTV do not show a Pareto front because their mean doses are almost constant (42-43Gy and 45-46Gy, respectively) for every combination of pitch and MF. Due to the major spatial overlap between oral mucosa and PTV, changes in pitch and MF can arbitrarily only slightly increase or decrease the mean dose. The latter is also true for the skin near PTV as the mean dose of this OAR is almost uninfluenced by a varying pitch or MF.

The Pareto fronts of the PTVs show two important effects. First, for pitches ≥0.30 the CI of PTV69 decreases/becomes better with increasing MF until the MF becomes higher than 2.6 or 2.8, depending on the used pitch. This sudden increase of the CI is due to a too high conformity of the plan. Because the CI looks for the 95 % isodose coverage of the PTV, the index becomes inferior when the PTV is completely covered by, for example, the 98 % isodose level. Secondly, the homogeneity of PTV56 is less than PTV69, because it depends on the location of the dose gradient. In these 2 dose level plans, the gradient can be placed either inside PTV56 or inside PTV69 as depicted in Fig. 2, or anywhere in between both extremes.

The Pareto fronts cover a wide range of Pareto optimal combinations: from pitch 0.10 and MF 3.0 to pitch 0.50 and MF 1.2. Despite the Pareto optimality of these points, not all of them result in clinically acceptable plans. The combination of a large pitch (0.50) and a low MF results in Pareto optimal combinations with clinically unacceptable doses to OAR. On the other side of the Pareto front, small pitches (0.10–0.20) introduce too much overlap between adjacent fan beams to be of any added value and consequently less modulation per rotation is required to end up with the same result. This excess of modulation leads to an unnecessary increase in monitor units and irradiation time, as confirmed in other studies [24, 25]. This Pareto front analysis shows that for a FW of 5 cm clinically acceptable combinations exist if pitch is chosen in a range of [0.23–0.45] and MF > 2.0. These values represent the best trade-offs between low OAR dose, PTV HI and CI and treatment time. The corresponding treatment time can vary from 4 m 05 s to 2 m 45 s.

The actual created Pareto fronts can be a good guideline in the planning of standard HT plans, increasing the intuitive understanding of how pitch and MF mutually interact with plan quality. However, patient-specific fine-tuning is still required in radiotherapy treatment planning. Because this study is not a complete multi-criteria optimization, plans superior to the shown Pareto fronts can be made by using a different optimization technique and/or a different delivery technique. Nevertheless, our study can help to minimize this time consuming fine-tuning and can serve as a planner’s reference in difficult cases.

## Conclusion

This study validates the vendor-recommended, default settings (pitch 0.430, MF ≈ 3.0 for FW 5 cm) as they fall in the trade-off range of most optimal combinations of pitch and MF and the REF5cm plan closely approximates the Pareto optimal fronts. However, by decreasing pitch there could be further improvement of plan quality (and increased treatment time) until pitch approaches ~0.23, with no reason to lower pitch beyond this value. As for every pitch a lower threshold for the corresponding MF is proposed where saturation in treatment time versus plan quality space occurs, plan quality only deteriorates without a gain in treatment time if MF is below this limit. It is up to the planner/radiation oncologist to decide what the best trade-off is along this curve within the range of [0.23–0.45] as long as MF > 2.0 for a FW of 5 cm.

As this study uses 5 oropharyngeal cancer patients to represent ‘the difficult case which best shows the possibilities of Helical Tomotherapy’, the authors believe the conclusions can be extrapolated towards other indications which require equal or less modulation.
